# Using a machine learning approach to predict outcome after surgery for degenerative cervical myelopathy

**DOI:** 10.1371/journal.pone.0215133

**Published:** 2019-04-04

**Authors:** Zamir G. Merali, Christopher D. Witiw, Jetan H. Badhiwala, Jefferson R. Wilson, Michael G. Fehlings

**Affiliations:** 1 Division of Neurosurgery, University of Toronto, Toronto, Ontario, Canada; 2 Division of Neurosurgery, St. Michael’s Hospital, Toronto, Ontario, Canada; 3 Division of Neurosurgery, Toronto Western Hospital, Toronto, Ontario, Canada; Leiden University Medical Center, NETHERLANDS

## Abstract

Degenerative cervical myelopathy (DCM) is a spinal cord condition that results in progressive non-traumatic compression of the cervical spinal cord. Spine surgeons must consider a large quantity of information relating to disease presentation, imaging features, and patient characteristics to determine if a patient will benefit from surgery for DCM. We applied a supervised machine learning approach to develop a classification model to predict individual patient outcome after surgery for DCM. Patients undergoing surgery for DCM as a part of the AOSpine CSM-NA or CSM-I prospective, multi-centre studies were included in the analysis. Out of 757 patients 605, 583, and 539 patients had complete follow-up information at 6, 12, and 24 months respectively and were included in the analysis. The primary outcome was improvement in the SF-6D quality of life indicator score by the minimum clinically important difference (MCID). The secondary outcome was improvement in the modified Japanese Orthopedic Association (mJOA) score by the MCID. Predictor variables reflected information about pre-operative disease severity, disease presentation, patient demographics, and comorbidities. A machine learning approach of feature engineering, data pre-processing, and model optimization was used to create the most accurate predictive model of outcome after surgery for DCM. Following data pre-processing 48, 108, and 101 features were chosen for model training at 6, 12, and 24 months respectively. The best performing predictive model used a random forest structure and had an average area under the curve (AUC) of 0.70, classification accuracy of 77%, and sensitivity of 78% when evaluated on a testing cohort that was not used for model training. Worse pre-operative disease severity, longer duration of DCM symptoms, older age, higher body weight, and current smoking status were associated with worse surgical outcomes. We developed a model that predicted positive surgical outcome for DCM with good accuracy at the individual patient level on an independent testing cohort. Our analysis demonstrates the applicability of machine-learning to predictive modeling in spine surgery.

## Introduction

Degenerative cervical myelopathy (DCM) is a spinal cord condition that results in progressive non-traumatic compression of the cervical spinal cord[1,2]. DCM is the most common cause of spinal cord dysfunction globally and can result in significant impairment in quality of life and function among affected patients[3]. Surgical decompression is the preferred treatment to alter the course of DCM and has been shown to improve functional outcome and quality of life in most but not all patients[4]. Indeed, the variability in extent of improvement in patients undergoing surgery for DCM is striking[4–8].

Selecting patients who will benefit from surgery for DCM necessitates consideration of a large quantity of information relating to disease presentation, imaging features, and patient characteristics. Previous studies have used classical regression models to associate pre-operative clinical factors with surgical outcome and identified predictors of a good surgical outcome[9–11]. Longer duration of DCM symptoms and more severe myelopathy have been identified as the most significant predictors of a worse surgical outcome[6,12].

Machine learning is an approach to data modeling that combines computer science and statistics with the goal of delivering maximal predictive accuracy. In recent years a number of studies have applied these new analytic tools to clinical databases to predict disease and treatment outcomes for conditions as varied as radiosurgery for arteriovenous malformations, childhood acute lymphoblastic leukemia, and subarachnoid hemorrhage[13–15]. These studies demonstrate that machine learning techniques can achieve higher predictive power and robustness than classical statistical methods.

In the present study our aim was to apply a supervised machine learning approach to develop a classification model to predict individual patient outcomes after surgery for DCM. A secondary aim was to use the machine learning approach to identify factors associated with a good surgical outcome.

## Materials and methods

### Patient population

We conducted a post-hoc analysis of 757 patients with DCM enrolled in the prospective, multi-center AOSpine CSM North America (CSM-NA; ClinicalTrials.gov NCT00285337) or AOSpine CSM International (CSM-I; ClinicalTrials.gov NCT00565734) cohort studies. The study received approval from the institutional review boards at the 12 participating sites (S2 Table). Patients were enrolled if they provided written consent and met eligibility criteria as follows: 1) age ≥ 18; 2) symptomatic DCM with one or more sign of myelopathy; 3) imaging evidence of cervical cord compression; and 4) no prior cervical spine surgery. Exclusion criteria were asymptomatic DCM, active infection, neoplastic disease, rheumatoid arthritis, trauma, ankylosing spondylitis, or concomitant lumbar stenosis. All enrolled patients underwent surgical decompression of the cervical spine, with or without an instrumented fusion.

### Surgical techniques

The surgical approach, number of operated levels, and use and type of instrumentation was at the discretion of the treating surgeon. Patients were treated anteriorly by cervical discectomy and/or corpectomy with fusion, or posteriorly, by laminectomy with or without instrumented fusion or laminoplasty, or by a combined circumferential approach.

### Baseline data and outcome measures

Baseline data included variables pertaining to patient demographics (e.g., age, sex, weight, height, race, education, etc.), clinical presentation (e.g., symptoms, signs, causative pathology, etc.), surgical treatment (e.g., approach, number of cervical levels operated on, operation duration, etc.), and detailed medical co-morbidities (previous MI, smoking history, cardiac pathology, psychiatric history, etc.). The pre-operative mJOA score, SF-36 score, neck disability index (NDI), and Nurick score were collected[16–19]. Our goal was to generate a model that could predict surgical outcome based on pre-operative clinical variables. We therefore did not include variables pertaining to the type of surgery (anterior vs. posterior) or the number of spinal levels operated on in our final model.

Outcome measures were assessed at 6-months, 12-months, and 24-months after surgery. The primary outcome measure was an improvement in quality of life as measured by the SF-6D score, derived from the SF-36 questionnaire. An improvement in quality of life was defined as an increase in the SF-6D score by 0.03, which represents the minimal clinically important difference (MCID)[20]. The secondary outcome measure was improvement in the mJOA score by at least 2 points, which represents the average MCID for all pre-operative disease severities[11,19].

### Data pre-processing and feature engineering

Missing data were handled in two ways. For features in which greater than 5% of data were missing the entire feature was eliminated. For features in which less than 5% of data were missing a k-nearest-neighbor algorithm (kNN) was used to impute the missing data. All samples were plotted in a 111-dimensional feature space and for each sample the 10 neighbors with the minimum Euclidian distance were identified. Missing values were then imputed by calculating the mean value among the 10 neighbors. Data pre-processing was carried out by creating dummy variables for categorical features and centering and scaling the ordinal and continuous features.

Feature selection was carried out using recursive feature elimination. A random forest model was generated with improvement in SF-6D as the outcome and the root mean squared error (RMSE) was recorded. The feature importance was determined by calculating the number of trees that used each feature and the most important feature was eliminated. Next the random forest model was generated with the remaining features and the process was continued iteratively until all features had been eliminated. The set of features that produced the lowest RMSE was chosen as the final feature set. The data sets were split into a training/validation and testing data set. The data were split such that class frequencies were equal between the training/validation and testing datasets.

### Model selection

Model selection, training, and testing was accomplished using RStudio**™** with the Caret package for machine learning functionality.

Initial model selection was carried out by comparing a random forest, support vector machine, logistic regression, simple decision tree, and artificial neural network (ANN) model using all features with improvement in SF-6D as the outcome. For initial model comparison 4-fold cross validation with two repeats was used. The default hyper-parameters provided by the Caret package were used for the random forest, support vector machine, logistic regression, decision tree, and artificial neural network models.

### Model training and testing

To train the final models repeated 10-fold cross validation with 5 repeats were used to minimize over-fitting[21]. Class imbalance was handled by up-sampling the under-represented class so that class frequencies were equal[22]. The number of random variables used at each node in the tree was designated (M-TRY). The model was tuned using a grid search strategy to vary M-TRY[21]. We used Area Under the Receiver Operating Characteristic (AUC) as the performance metric to compare models.

## Results

### Data pre-processing

Of the 757 patients a varying number were excluded for incomplete follow-up information leaving 605 patients with 6-month follow-up, 583 with 12-month follow-up, and 539 with 24-month follow-up. Baseline characteristics for the 6-month follow-up dataset can be seen in (Table 1).

**Table 1:** Baseline characteristics of combined training, validation, and testing dataset.

	n = 605
**Age (IQR)**	56 (48,64)
**Male**	62.7%
**Current Smoker**	26.8%
**Comorbidities**	
*Previous MI*	3.7%
*Angina*	6.7%
*Congestive Heart Failure*	0.9%
*Cardiac Arrhythmia*	2.2%
*Hypertension*	38.6%
*Peripheral Arterial Disease*	1.5%
*Respiratory Disease*	9.1%
*Hepatic Disease*	2.2%
*Gastrointestinal Disease*	12.4%
*Pancreatic Disease*	0%
*Diabetes*	13.3%
*Psychiatric Disease*	11.0%
*Rheumatic Disease*	4.5%
*Previous Stroke*	2.0%
*Neuromuscular Disease*	2.1%
**Diagnosis**	
*Disk Herniation*	71.7%
*Spondylosis*	76.9%
*OPLL*	21.0%
*HLF*	24.4%
*Subluxation*	5.7%
**Symptoms**	
*Numb Hands*	88.8%
*Clumsy Hands*	74.1%
*Gait Difficulty*	75.2%
*Bilateral Arm Paresthesia*	56.5%
*L’Hermitte’s Parasthesias*	26.6%
*Weakness*	82.3%
**Signs**	
*Corticospinal Distribution of Motor Deficits*	62.4%
*Atrophy of Hand Intrinsic Muscles*	35.8%
*Hyperreflexia*	77.4%
*Hoffman’s Reflex*	62.0%
*Babinski Reflex*	35.3%
*Lower Limb Spasticity*	46.6%
*Unstable Gait*	58.4%

A varying number of dummy variables were created for the categorical features such that all categorical features had only two classes. The pre-operative insurance information had greater than 5% missing values and the features pertaining to insurance were therefore eliminated. All other features had either none or less than 5% missing values. Next a k-nearest-neighbor algorithm was used to impute missing values in the remaining features. Finally, the ordinal and continuous variables were centered and scaled to a mean of 0 and standard deviation of 1. This left 111 features to be carried forward into model selection and feature engineering.

### Model selection

Model selection was carried out with all 111 features and the outcome was improvement in the SF-6D score. A random forest, support vector machine, logistic regression, simple decision tree, and artificial neural network model was trained using the 6-month, 12-month, and 24-month datasets. The fit and performance of the four models was compared in (Table 2). The random forest model exhibited the best performance at all time-points with an area under the receiver operating characteristic curve (AUC) of 0.64, 0.68, and 0.7 at 6-months, 12-months, and 24-months respectively. The random forest model also exhibited the best predictive performance at all time-points with an accuracy of 70%, 71%, and 69% at 6-months, 12-months, and 24-months respectively. The random forest model was thus chosen for further optimization.

**Table 2:** Comparison of model performance when predicting improvement in SF6D score.

	AUC			Accuracy		
	6 months	12 months	24 months	6 months	12 months	24 months
Random Forest	0.64	0.68	0.7	0.70	0.71	0.69
Support Vector Machine	0.65	0.62	0.7	0.64	0.67	0.68
Logistic Regression	0.58	0.63	0.67	0.62	0.60	0.65
Decision Tree	0.65	0.63	0.67	0.64	0.49	0.65
Artificial Neural Network	0.59	0.52	0.53	0.56	0.52	0.51

### Feature selection

**Fig 1:**
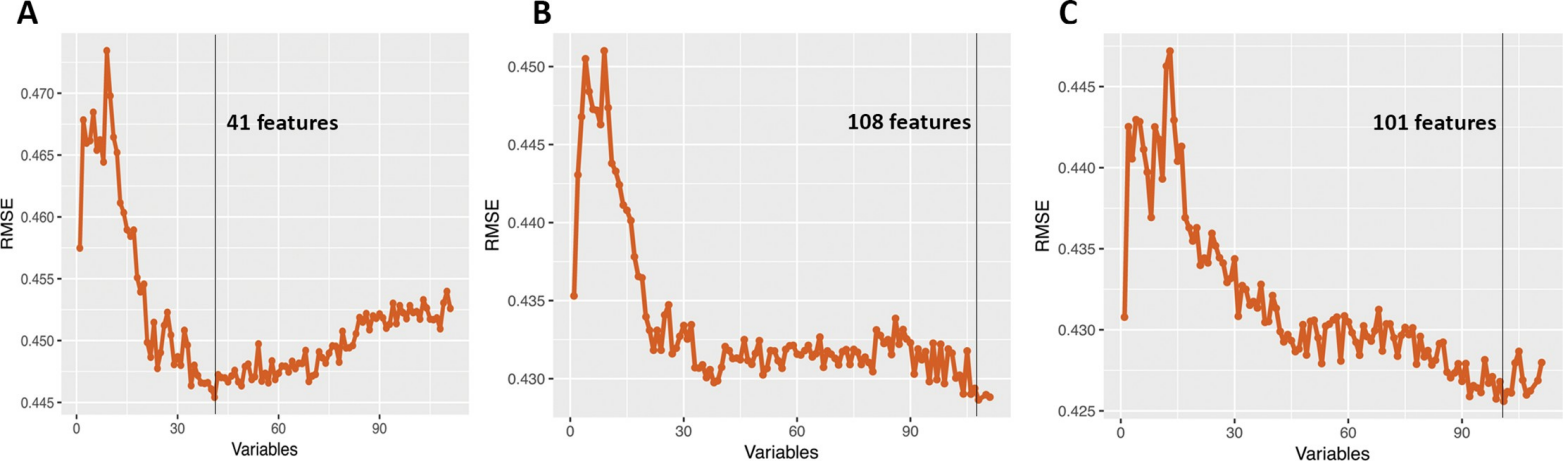
Results of the recursive feature elimination algorithm applied to 6-month follow-up (A), 12-month follow-up (B), and 24-month follow-up (C). The figures demonstrate the change in root mean squared error (RMSE) as features were iteratively added to the model. As greater number of features were added to the model the RMSE decreased to a minimum value, demonstrating best model fit, then began to increase as greater numbers of ‘distracting’ features were added. The set of features that achieved the minimum RMSE were used for model training (shown by vertical black line).

The recursive feature elimination algorithm was run using all 111 features and improvement in the SF-6D score as the outcome (Fig 1). The feature set that produced the lowest RMSE was chosen for model training and all other features were eliminated. This process of feature selection resulted in 41 features for the 6-month dataset, 108 features for the 12-month dataset, and 101 features for the 24-month dataset (S1 Table).

### Model training and testing

**Fig 2:**
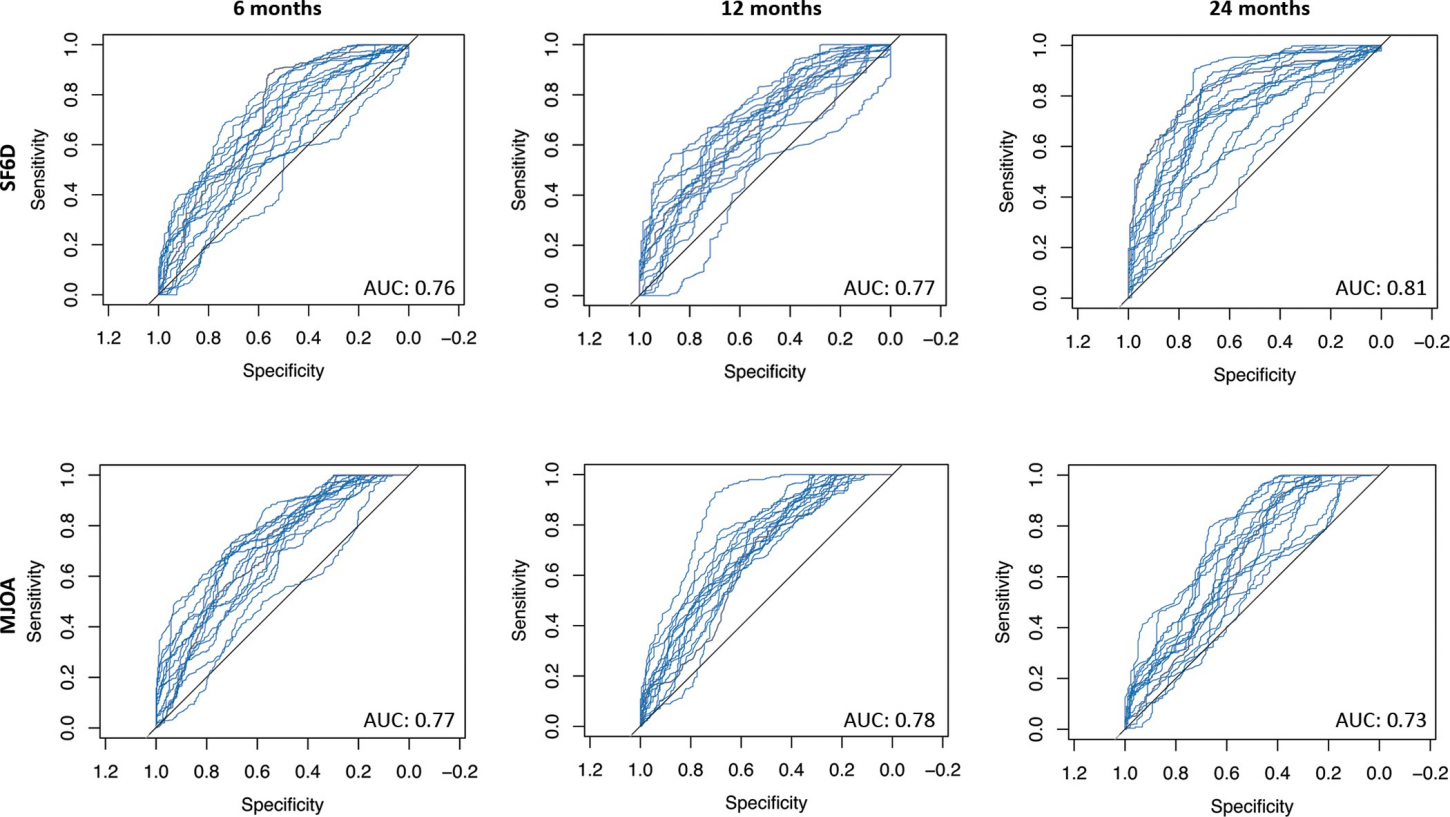
Receiver operating characteristic curves for the random forest model at all follow-up points on the training/validation dataset. The blue lines represent each cross validation fold.

The dataset was split with 70% of samples assigned to the training/validation dataset and 30% to the testing dataset. A separate random forest model was trained using the selected features for each follow-up time-point. At each follow-up time-point a separate model was trained with improvement in SF-6D score and mJOA score as outcomes. The model fit during each cross-validation run is summarized in (Fig 2). Models were tuned automatically using a grid search strategy and the best performing model was chosen from the entire set of generated models. The final random forest model had M-TRY of 9, 37, and 35 for the 6-month, 12-month, and 24-month time-points, respectively. All random forest models used 500 trees with a tree depth of 20.

The best performing model for each time-point and outcome was tested on the testing dataset. Model performance is summarized in (Table 3). The best performance was achieved at the 12-month time-point with a accuracy of 77.0% and 71.3% when predicting improvement in the SF-6D and mJOA score respectively. At other time-points the model performance was comparable with a accuracy range of (67.7% - 77.0%) and an AUC range of 0.68–0.73). Confusion matrixes were generated for the testing data (Table 4).

**Table 3:** Predictive performance of the random forest model on the testing dataset.

	Samples	Features	Accuracy	Sensitivity	Specificity	PPV	NPV	AUC
SF-6D								
6 months	181	41	71.8%	0.75	0.50	0.90	0.25	0.71
12 months	181	108	77.0%	0.78	0.63	0.98	0.12	0.70
24 months	181	101	70.8%	0.74	0.47	0.92	0.17	0.73
mJOA								
6 months	195	41	66.7%	0.70	0.59	0.82	0.43	0.73
12 months	188	108	71.3%	0.72	0.69	0.91	0.36	0.73
24 months	168	101	64.9%	0.63	0.80	0.96	0.23	0.67

**Table 4:** Confusion matrix showing the random forest model predictions for the independent testing dataset at 6, 12, and 24 months.

6 months			
Prediction Reference	Not Improved	Improved	Totals
Not Improved	13	13	26
Improved	38	117	156
Totals	51	130	
12 months			
Prediction Reference	Not Improved	Improved	Totals
Not Improved	5	3	8
Improved	37	129	166
Totals	42	132	
24 months			
Prediction Reference	Not Improved	Improved	Totals
Not Improved	8	9	17
Improved	38	106	144
Totals	46	115	

### Feature importance

**Fig 3:**
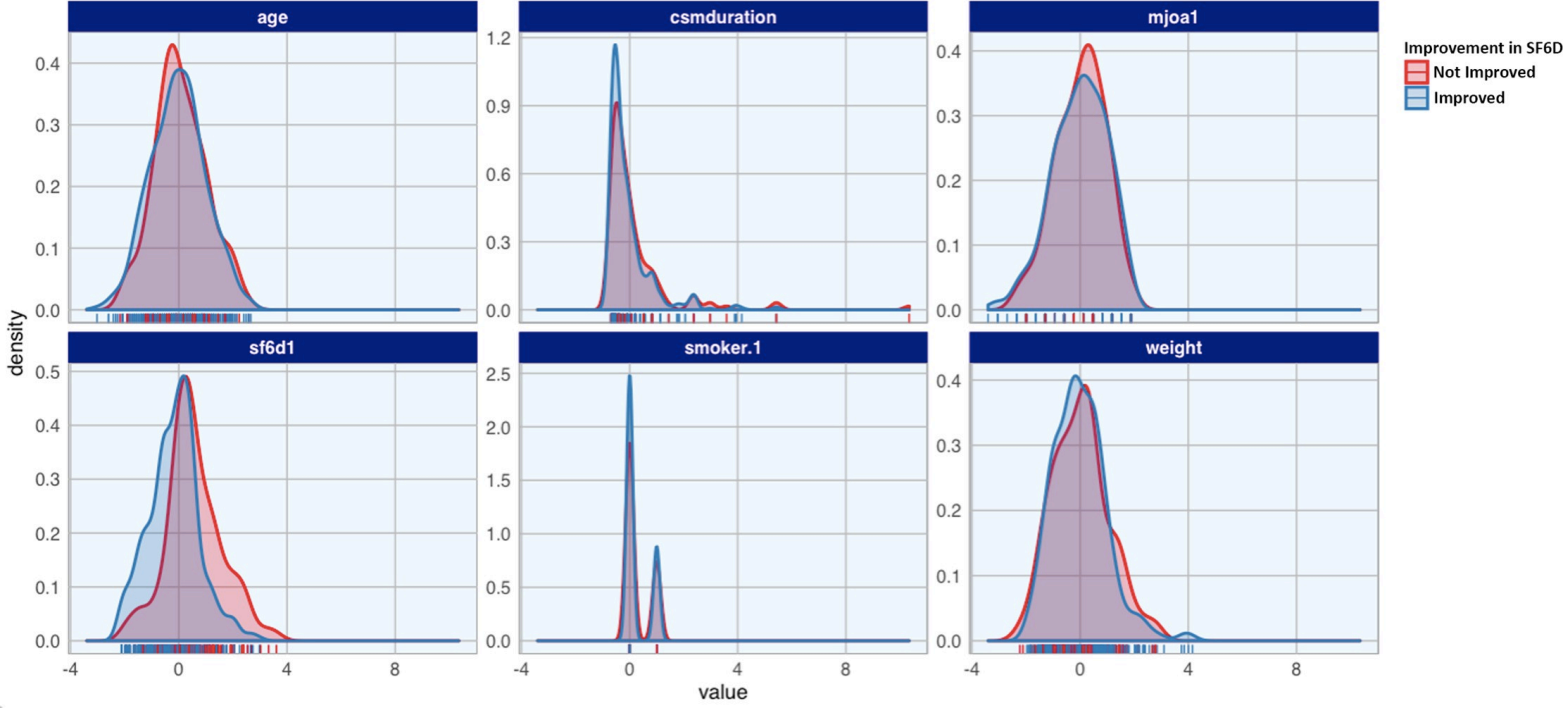
Density plots for the top 6 most important predictive features selected by the random forest model. These density plots demonstrate the distribution of the key features between the patients who did (blue) and did not (red) show improvement in SF6D at 1-year follow-up. In all key features there is overlap of the curves, demonstrating that there is no one singe feature that can alone predict if a patient with DCM will improve with surgery.

The random forest models were analyzed to determine the features that were the most important for prediction of the outcome. The most important features varied slightly between the models for the different time-points. However, the following 6 features were ranked among the 10 most important features at all time-points and for both outcome measures: age, duration of DCM symptoms, pre-operative mJOA score, pre-operative SF-6D score, current smoker, body weight. The distribution of these top 6 features is summarized in (Fig 3).

## Discussion

Surgical decompression is the preferred treatment for DCM and can result in long-term improvement of myelopathic symptoms and quality of life in the majority of patients although the extent of improvement can vary widely[4]. In this study we applied a machine learning approach to a multi-centre prospective database and were able to predict outcome after surgery for DCM at the individual patient level with good performance. In addition we identified the following pre-operative variables as important predictors of surgical outcome: older age, duration of DCM symptoms, pre-operative disease severity, body weight, and smoking status. To our knowledge this is the first study to apply a machine learning approach to predict surgical outcome after DCM. These results can be applied to guide surgical decision-making and support the results of previous studies using classical statistical methods.

In our initial analysis we compared a random forest (RF), support vector machine (SVM), logistic regression (LR), simple decision tree (DT), and artificial neural network (ANN) model on the entire feature set. The RF and SVM models outperformed the LR, DT, and ANN models. These results are similar to other studies that found that RF and SVM models outperform classical LR and DT models on classification tasks on large health datasets[13,14]. This is attributable to the ability of the RF and SVM models to model complex non-linear and conditional relationships that may be missed by the LR and DT models. Of note, the ANN performed poorly compared to the other tested models. This is likely due to the limited number of training samples that were available to train the ANN. ANN models generally require a higher number of training samples than SVM or RF models for adequate training [13]. The RF model outperformed the SVM at all time-points. This is likely due to the ability of the RF model to avoid over-fitting on datasets with a low sample to feature ratio. Our dataset had a sample to feature ratio of approximately 5:1, which may have limited the ability of the SVM model to converge on a local minimum. It is possible that a technique of dimensionality reduction, such as principle component analysis, would have increased the predictive performance of the SVM model. A low ratio of samples to features is a common challenge encountered in health datasets and RF models are thus well suited to classification tasks in this domain.

The RF model was then optimized using a process of feature selection and 10-fold cross validation. When tested on the independent testing cohort of 180 patients the final RF model identified patients who would benefit from surgery with a sensitivity of 75%, 78%, and 74% and AUC of 0.71, 0.73, and 0.70 at 6, 12, and 24 months respectively. Given the complexity of the pathology and patient cohort this is a good sensitivity and is comparable to what has been achieved by machine learning models applied to other health datasets. On the validation cohort our model achieved a higher AUC of 0.85, 0.83, and 0.87 at 6, 12, and 24 months respectively. It is generally accepted that classification models will exhibit a certain degree of over-fitting on the validation cohort. It is thus important to note that our model exhibited good sensitivity and AUC on the testing cohort, which suggests our model is generalizable to a broader patient population.

Comparison of our RF model with previously published regression models is limited due to differences in methodology. In addition, previously published models did not utilize an independent testing cohort to evaluate model performance and may therefore be susceptible to over-fitting. A previously published model utilized a logistic regression to predict surgical outcome at 12 months[9,23]. This model achieved an AUC of 0.74 on the validation cohort, while our model achieved an AUC of 0.83 at the same time-point on the validation cohort. This previously published model was not tested on an independent testing cohort and a full comparison with our RF model is thus not possible. In addition this model defined a good surgical outcome as a post-operative mJOA score ≥ 16 at 12-months, while we defined a good surgical outcome as an improvement in the SF-6D or mJOA by the MCID, which further limits comparison. Nonetheless, our random forest model appears to outperform previously published regression models.

While our model exhibited good sensitivity the specificity was moderate at 0.5, 0.47, and 0.63 at 6, 12, and 24 months respectively. This indicates that while our model was able to identify the majority of patients who benefit from surgery, it misclassified patients who did not benefit from surgery approximately 50% of the time. This is likely due to the fact that the majority (75–78%) of patients in the overall cohort benefited from surgery. Given the relatively low number of patients in the cohort who did not benefit from surgery there were a limited number of samples with a negative outcome to be used for model training. We attempted to account for this class imbalance by using up-sampling. It is likely that a larger cohort of patients with a negative surgical outcome would be required to further increase the performance of our model.

Our RF model identified longer duration of DCM symptoms, worse pre-operative disease severity, higher age, greater body weight, and current smoking status as being associated with worse surgical outcomes. These results support the findings of previously published models and expert consensus[24–28]. A previously published logistic regression model identified higher age, longer duration of DCM symptoms, current smoking status, psychiatric comorbidities, and gait impairment as being associated with worse surgical outcome, which is similar to the results of our model[10,29]. A recent systematic review again identified worsened pre-operative disease severity and longer duration of DCM symptoms as being associated with worsened surgical outcome[12]. In summary our model using a machine learning approach identified similar factors as being associated with surgical outcomes as previous models that used classical statistical methods.

Our analysis addresses a number of limitations of previous studies. Our use of a machine learning approach allowed us to model complex non-linear and conditional relationships, avoid over-fitting, account for the non-normal distribution of outcomes, and generate individual patient-level predictions. Our model thus achieved better performance than previously published models. In addition our model demonstrated good performance on an independent patient cohort that wasn’t used for model training, which suggests our model is generalizable to a broader patient population. Despite these strengths our study is subject to some limitations. Firstly, approximately 29% of patients were lost to follow-up by the 24-month time-point. Secondly, our model used pre-operative clinical variables relating to disease presentation, patient demographics, and medical comorbidities. We did not, however, include radiographic parameters when training our model, as this information was not available for the majority of the patients in our cohort. Our model would likely have performed better if features relating to pre-operative magnetic resonance images (MRIs) had been included in model training[30,31]. Finally, we were limited by the number of samples in our dataset. Although we found we had enough samples to train a binary RF classification model with good accuracy, we did not have sufficient samples to generate a multi-class model. In addition, we did not have sufficient samples to train an ANN, which may have limited the predictive power of our final model. These limitations highlight the importance of a large diverse dataset when attempting to create a clinical prediction model. Although machine learning provides a powerful toolset to model complex patterns and generate predictions, machine learning models require relatively large datasets to achieve optimum performance when compared to traditional statistical methods. Nonetheless, our model was able to address an important clinical endpoint–improvement of the mJOA score and SF-6D score by the MCID.

## Conclusion

We retrospectively applied a machine learning approach to a multi-centre cohort of patients who underwent surgical decompression for DCM. Our final random forest model was able to predict positive surgical outcome with good accuracy at the independent patient level on an independent testing cohort that was not used for model training. Our model identified worse pre-operative disease severity, longer duration of DCM symptoms, older age, higher body weight, and current smoking status as being associated with worse surgical outcomes. To our knowledge our model, using a machine learning approach, achieved a higher accuracy than previously published models. We identified longer duration of DCM symptoms, worse pre-operative disease severity, higher age, higher body weight, and current smoking status as being associated with worse surgical outcomes, which supports the results of previous studies. Our analysis demonstrates the applicability of machine-learning to predictive modeling in spine surgery.

## Supporting information

S1 Table List of variables included in the final random forest model at 6-month, 12-month, and 24-month time-points with relative importance of each variable. (DOCX)

S2 Table Overview of institutional review boards involved in NCT00285337, NCT00565734 clinical trials. (DOCX)

## Abbreviations

MRI: Magnetic Resonance Imaging
DCM: Degenerative Cervical Myelopathy
MCID: Minimum Clinically Important Difference
mJOA: Modified Japanese Orthopedic Association
AUC: Area Under the Receiver Operating Characteristic Curve
NDI: Neck Disability Index
kNN: k Nearest Neighbor
RMSE: Root Mean Squared Error
RF: Random Forest
SVM: Support Vector Machine
LR: Logistic Regression
DT: Decision Tree
