# Supplementary material for: Using a machine learning approach to predict outcome after surgery for degenerative cervical myelopathy
Source: PLoS One. 2019 Apr 4;14(4):e0215133. doi: 10.1371/journal.pone.0215133 (PMC6448910; doi:10.1371/journal.pone.0215133)
Supplement: S1 Table — (DOCX) [file pone.0215133.s001.docx]

Table S1 – List of variables included in the final random forest model at 6-month, 12-month, and 24-month time-points with relative importance of each variable.

| 6 month follow up | | 12 month follow up | | 24 month follow up | |
| --- | --- | --- | --- | --- | --- |
| Variable | Relative Importance | Variable | Relative Importance | Variable | Relative Importance |
| mjoa1 | 100 | mjoa1 | 100 | mjoa1 | 100 |
| ossifiedligament.1 | 12.13727309 | race.other | 5.097397818 | csmduration | 33.82716997 |
| nurick1 | 26.15053385 | sf6d1 | 27.46914563 | htngrade.severe | 0.303509799 |
| sf6d1 | 55.55428608 | nurick1 | 12.13195718 | education.four | 3.166715055 |
| csmduration | 53.69384529 | age | 30.79875318 | diabetesgrade.none | 3.572028171 |
| ndi1 | 53.56542871 | oplevels.3 | 4.744146566 | age | 38.13914383 |
| broadgait.1 | 9.541430053 | respiratorygrade.none | 2.923276698 | race.white | 5.330261366 |
| education.none | 3.7463883 | race.white | 3.927182413 | anginagrade.none | 3.062808932 |
| chfgrade.none | 1.198593733 | arrythmiagrade.none | 2.077234291 | sf6d1 | 30.39913959 |
| race.other | 5.050707051 | smoker.1 | 5.487198162 | nurick1 | 13.67049981 |
| weight | 70.88290091 | chfgrade.moderate | 0.17801971 | respiratorygrade.severe | 2.963884943 |
| oplevels.4 | 11.76586939 | weight | 33.38101386 | oplevels.5 | 5.46067772 |
| age | 59.33649544 | csmduration | 23.17379963 | oplength | 40.16509905 |
| education.thirteen | 12.95692425 | ndi1 | 27.19983277 | ndi1 | 30.99988702 |
| arrythmiagrade.none | 5.183445302 | education.none | 4.30766473 | spastic.1 | 6.724553305 |
| impairedgait.1 | 7.615917704 | smokerhx.1 | 4.747683597 | ossifiedligament.1 | 4.917173087 |
| oxygen.1 | 0.542978377 | respiratorygrade.severe | 0.442248621 | race.other | 2.756890463 |
| education.four | 5.531705681 | impairedgait.1 | 3.05808332 | marital.widowed | 2.756980758 |
| clumbsyhands.1 | 9.752336914 | broadgait.1 | 3.405090988 | hepaticgrade.none | 2.588320632 |
| cardiac | 13.26669 | spondy.1 | 4.236241227 | oplevels.4 | 5.092726281 |
| marital.widowed | 4.727578755 | chfgrade.none | 0.992768506 | smokerhx.1 | 4.055164755 |
| psychiatricgrade.moderate | 1.467437197 | rheumgrade.moderate | 0.910790118 | renalgrade.moderate | 0.30085736 |
| strokegrade.moderate | 0.961174247 | oplevels.4 | 4.788054447 | impairedgait.1 | 9.368515899 |
| respiratorygrade.none | 5.652582101 | education.four | 2.394346201 | oplevels.6 | 1.615444338 |
| marital.divorced | 5.376414827 | gender.1 | 3.447708179 | esrdgrade.none | 0.140632162 |
| spondy.1 | 9.212824254 | height | 26.47827215 | ligamenthypertrophy.1 | 3.97770233 |
| respiratorygrade.moderate | 3.261561053 | psychiatricgrade.none | 1.941816763 | weakness.1 | 4.770291723 |
| hepaticgrade.severe | 0.34956585 | marital.married | 4.267885365 | arrythmiagrade.moderate | 0.486445495 |
| smokerhx.1 | 11.09970419 | renalgrade.moderate | 0.380569982 | respiratorygrade.moderate | 2.154056292 |
| impairedmotor.1 | 11.9973589 | anginagrade.none | 1.418578913 | psychiatricgrade.moderate | 1.518143832 |
| peripheralarterialgrade.none | 0.523222534 | weakness.1 | 2.706190862 | psychiatricgrade.none | 4.393178257 |
| venousgrade.moderate | 0.466461349 | clumbsyhands.1 | 4.030255387 | migrade.none | 0.618836188 |
| htngrade.severe | 1.000677059 | hoffman.1 | 4.583569237 | clumbsyhands.1 | 4.767681335 |
| marital.married | 8.553194861 | esrdgrade.none | 0.108509958 | congenital.1 | 1.934194458 |
| spastic.1 | 10.8026625 | intestinalgrade.moderate | 0.654880633 | anginagrade.moderate | 0.596512618 |
| oplevels.5 | 7.871224271 | htngrade.none | 3.454933192 | arrythmiagrade.none | 1.667406555 |
| arrythmiagrade.severe | 0 | oplength | 29.96410059 | smoker.1 | 9.615721207 |
| intestinalgrade.none | 5.256333457 | respiratorygrade.moderate | 1.210188931 | intestinalgrade.none | 3.319680747 |
| race.white | 8.811349659 | venousgrade.moderate | 0.612017692 | rheumgrade.none | 2.671470768 |
| peripheralarterialgrade.moderate | 0.607508682 | oplevels.5 | 2.478410547 | marital.married | 5.096016821 |
| hepaticgrade.none | 2.066066939 | oplevels.2 | 2.323613724 | bilateralsensory.1 | 5.172355678 |
|  |  | cardiac | 3.585487905 | spondy.1 | 4.318634625 |
|  |  | impairedmotor.1 | 3.913618343 | rheumgrade.moderate | 0.449330819 |
|  |  | htngrade.moderate | 2.171909502 | lhermitte.1 | 4.340683322 |
|  |  | peripheralarterialgrade.none | 0.552507175 | hoffman.1 | 5.057211707 |
|  |  | handatrophy.1 | 5.255790649 | broadgait.1 | 4.485900348 |
|  |  | plantar.1 | 3.276884996 | respiratorygrade.none | 3.631644816 |
|  |  | marital.single | 2.111648842 | education.none | 1.598307156 |
|  |  | diabetesgrade.moderate | 0.980590559 | height | 29.48391516 |
|  |  | htngrade.severe | 0.613558925 | peripheralarterialgrade.none | 0.229071374 |
|  |  | othersource.1 | 0.587624696 | peripheralarterialgrade.moderate | 0.033427188 |
|  |  | marital.widowed | 1.845552476 | plantar.1 | 4.521474741 |
|  |  | oplevels.6 | 1.617959567 | education.other | 0.801801635 |
|  |  | numbhands.1 | 2.458316313 | venousgrade.moderate | 0.674637479 |
|  |  | oxygen.1 | 0.115836142 | education.twelve | 5.180387842 |
|  |  | hyperreflexia.1 | 2.893469186 | oplevels.2 | 3.987516359 |
|  |  | venousgrade.none | 2.852238733 | weight | 38.14521464 |
|  |  | disk.1 | 4.342495984 | hyperreflexia.1 | 4.298887412 |
|  |  | migrade.moderate | 0.618734526 | strokegrade.none | 1.557929281 |
|  |  | race.native | 0.952000459 | htngrade.none | 3.882655405 |
|  |  | arrythmiagrade.severe | 0.120401642 | marital.single | 3.400517763 |
|  |  | rheumgrade.none | 0.956513096 | impairedmotor.1 | 3.811178648 |
|  |  | anginagrade.moderate | 0.851315551 | education.thirteen | 3.426062786 |
|  |  | education.twelve | 3.456386259 | gender.1 | 3.671370088 |
|  |  | anginagrade.severe | 0.014298186 | oplevels.3 | 5.15438463 |
|  |  | neuromusculargrade.none | 1.508131656 | venousgrade.none | 1.484548066 |
|  |  | peripheralarterialgrade.moderate | 0.668345975 | race.native | 0.050140475 |
|  |  | hepaticgrade.severe | 0.013172658 | neuromusculargrade.none | 5.532596716 |
|  |  | education.thirteen | 4.314550053 | hepaticgrade.severe | 0.051036376 |
|  |  | subluxation.1 | 1.333625653 | previousop.1 | 0.043608014 |
|  |  | education.other | 1.39223544 | chfgrade.none | 0.687799825 |
|  |  | ligamenthypertrophy.1 | 3.258474075 | intestinalgrade.moderate | 0.120841751 |
|  |  | chfgrade.severe | 0 | cardiac | 3.64436639 |
|  |  | diabetesgrade.severe | 0.019421065 | diabetesgrade.moderate | 0.845813486 |
|  |  | esrdgrade.moderate | 0 | marital.divorced | 2.820015262 |
|  |  | esrdgrade.severe | 0 | chfgrade.severe | 0 |
|  |  | hepaticgrade.moderate | 0.006499175 | diabetesgrade.severe | 0 |
|  |  | intestinalgrade.severe | 0 | esrdgrade.moderate | 0.033298934 |
|  |  | neuromusculargrade.severe | 0.148782054 | esrdgrade.severe | 0 |
|  |  | pacemaker.1 | 0.202236982 | hepaticgrade.moderate | 0 |
|  |  | pancreasgrade.none | 0.006499175 | intestinalgrade.severe | 0 |
|  |  | paralysisgrade.moderate | 0 | migrade.severe | 0 |
|  |  | paralysisgrade.none | 0.100218716 | neuromusculargrade.moderate | 0.460316214 |
|  |  | paralysisgrade.severe | 0 | neuromusculargrade.severe | 0.348177359 |
|  |  | peripheralarterialgrade.severe | 0.049406775 | oxygen.1 | 0.055704184 |
|  |  | psychiatricgrade.severe | 0.036434375 | pacemaker.1 | 0.513352538 |
|  |  | race.ocean | 0 | pancreasgrade.none | 0.020595893 |
|  |  | rheumgrade.severe | 0.008123969 | paralysisgrade.moderate | 0.013044065 |
|  |  | strokegrade.moderate | 0.499251944 | paralysisgrade.severe | 0 |
|  |  | strokegrade.severe | 0.031177051 | peripheralarterialgrade.severe | 0.354579898 |
|  |  | venousgrade.severe | 0 | psychiatricgrade.severe | 0.038154712 |
|  |  | strokegrade.none | 0.456396185 | race.ocean | 0 |
|  |  | lhermitte.1 | 3.028027799 | rheumgrade.severe | 0.038617299 |
|  |  | congenital.1 | 2.635325226 | strokegrade.moderate | 0.855607848 |
|  |  | diabetesgrade.none | 1.977870559 | strokegrade.severe | 0 |
|  |  | neuromusculargrade.moderate | 0.563113112 | venousgrade.severe | 0 |
|  |  | race.black | 1.112979862 | disk.1 | 3.360604089 |
|  |  | intestinalgrade.none | 2.217038519 | arrythmiagrade.severe | 0 |
|  |  | marital.other | 1.226742993 | oplevels.7 | 0.022426639 |
|  |  | arrythmiagrade.moderate | 0.374614406 | paralysisgrade.none | 0.022688022 |
|  |  | bilateralsensory.1 | 3.654689023 | subluxation.1 | 2.051526734 |
|  |  | previousop.1 | 0.742182863 |  |  |
|  |  | spastic.1 | 3.689589785 |  |  |
|  |  | psychiatricgrade.moderate | 1.122839964 |  |  |
|  |  | marital.divorced | 2.337990547 |  |  |
|  |  | migrade.severe | 0 |  |  |
|  |  | hepaticgrade.none | 1.297991639 |  |  |
|  |  | migrade.none | 0.693395723 |  |  |
