# Supplementary material for: Using a machine learning approach to predict outcome after surgery for degenerative cervical myelopathy
Source: PLoS One. 2019 Apr 4;14(4):e0215133. doi: 10.1371/journal.pone.0215133 (PMC6448910; doi:10.1371/journal.pone.0215133)
Supplement: S2 Table — (DOCX) [file pone.0215133.s002.docx]

Table S2 – Overview of institutional review boards involved in NCT00285337, NCT00565734 clinical trials.

| Location | Institution(s) |
| --- | --- |
| Thornton, Colorado, United States, 80229 | Spine Education and Research Institute, Research Ethics Board |
| Atlanta, Georgia, Unites States, 30329 | Emory University, Office of Research Compliance |
| Indianapolis, Indiana, United States, 46260 | Indiana Spine Group, Institutional Review Board |
| Kansas City, Kansas, United States, 66160 | Kansas University Medical Center, Research Institute |
| Baltimore, Maryland, United States, 21287 | John Hopkins University, Institutional Review Board |
| Boston, Massachusetts, United States, 02115 | Brigham and Women’s Hospital, Partners Human Research Committee  New England Baptist Hospital, Institutional Review Board |
| Rochester, Minnesota, United States, 55905 | Mayo Clinic, Institutional Review Board |
| Philadelphia, Pennsylvania, United States, 19107 | Thomas Jefferson University and Rothman Institute Orthopaedics |
| Salt Lake City, Utah, United States, 84108 | University of Utah, Institutional Review Board |
| Charlottesville, Virginia, United States, 22908 | University of Virginia, Institutional Review Board |
| Seattle, Washington, United States, 98104 | Harborview Medical Center, Institutional Review Board |
| Toronto, Ontario, Canada, M5T2S8 | University of Toronto University Health Network, Research Ethics Board |
